# Matrix-free human 2D organoids recapitulate duodenal barrier and transport properties

**DOI:** 10.1186/s12915-024-02105-7

**Published:** 2025-01-05

**Authors:** Kopano Valerie Masete, Dorothee Günzel, Jörg-Dieter Schulzke, Hans-Jörg Epple, Nina A. Hering

**Affiliations:** 1 Clinical Physiology/Nutritional Medicine, Medical Department, Division of Gastroenterology, Infectiology and Rheumatology, Charité - Universitätsmedizin Berlin, Campus Benjamin Franklin, Berlin, Germany; 2 Department of Gastroenterology, Rheumatology and Infectious Diseases, Charité - Universitätsmedizin Berlin, Campus Benjamin Franklin, Berlin, Germany; 3 Antibiotic Stewardship Team, Medical Directorate, Charité - Universitätsmedizin Berlin, Campus Benjamin Franklin, Berlin, Germany; 4 Department of General and Visceral Surgery, Campus Benjamin Franklin, Charité - Universitätsmedizin Berlin, Hindenburgdamm 30, Berlin, 12203 Germany

**Keywords:** Matrigel-free, Barrier function, Organoids, Small intestine, 3Rs Principle

## Abstract

**Background:**

Traditionally, transformed cell line monolayers have been the standard model for studying epithelial barrier and transport function. Recently, intestinal organoids were proposed as superior in recapitulating the intestine. Typically, 3D organoids are digested and seeded as monolayers on gelatinous matrix pre-coated surfaces for anchorage. As this coat could potentially act as a diffusion barrier, we aimed to generate robust human duodenum-derived organoid monolayers that do not need a gelatinous matrix for anchorage to improve upon existing models to study epithelial transport and barrier function.

**Results:**

We characterized these monolayers phenotypically regarding polarization, tight junction formation and cellular composition, and functionally regarding uptake of nutrients, ion transport and cytokine-induced barrier dysfunction. The organoid monolayers recapitulated the duodenum phenotypically as well as functionally regarding glucose and short-chain fatty acid uptake. Tumour necrosis factor-alpha induced paracellular transport of 4-kDa Dextran and transcytosis of 44-kDa horseradish peroxidase. Notably, forskolin-stimulated chloride secretion was consistently lower when organoid monolayers were seeded on a layer of basement membrane extract (BME).

**Conclusions:**

BME-free organoid monolayers represent an improved model for studying transcytotic, paracellular but especially transcellular transport. As BME is extracted from mice, our model furthers efforts to make organoid culture more animal-free.

**Supplementary Information:**

The online version contains supplementary material available at 10.1186/s12915-024-02105-7.

## Background

The small intestinal epithelium is an important physical barrier preventing microbial pathogens from entering the body while allowing the controlled absorption of nutrients. The duodenal epithelial monolayer is made up of mostly enterocytes, Paneth, goblet, stem and enteroendocrine cells. Intestinal epithelial cells are polarized, with the apical side of cells being held together by the apical junctional complex including tight junctions, which maintain the epithelial barrier and regulate paracellular transport of ions and water. The intestinal epithelial barrier can be disrupted by several cytokines including tumour necrosis factor-alpha (TNF-α), a well-characterized inducer of epithelial cell apoptosis and tight junction disruptions [1–4]. These disruptions cause dysregulated exposure of immune cells to pathogenic luminal content, contributing to the disease progression of inflammatory bowel disease (IBD) [5, 6] and untreated human immunodeficiency virus (HIV) infection [7, 8]. There is therefore an urgent need for in vitro models that could accurately recapitulate these processes, to better study intestinal barrier in health and disease.

Traditionally, models to study the intestinal barrier have been limited to animal models, transformed cell lines and human primary tissue. While animal models provide comprehensive in vivo systemic context, species-specific differences limit their translatability. Although transformed cell lines are derived from humans and are easy to culture, they contain tumour-associated mutations, show aberrant gene expression patterns and do not contain all the cell types found in the intestine [9–11]. Furthermore, they derive from one individual and thus cannot capture human-to-human heterogeneity. Human primary tissue can be used as an alternative as it more closely represents in vivo conditions. However, primary tissue is difficult to culture and passage, requiring constant access to human samples. To address these problems, long-term expandable cultures were generated from mouse and human tissue-derived stem cells for the first time in 2009 and 2011, respectively [12, 13], giving rise to a new in vitro model system termed “organoids”.

Organoid research is an important step towards animal-free research, in line with the 3Rs Principle, referring to the replacement, reduction and refinement of animal experiments [14]. Unlike cell lines, organoids recapitulate the heterogeneous cellular composition of the tissue from which they originate. Given the appropriate stem cell niche growth factor cocktail and a gelatinous matrix scaffold such as basement membrane extract (BME) or collagen, adult stem cells self-organize into mini 3D organ-like structures in vitro [15]. 3D organoids have yielded vital information in disease modelling, developmental biology, regenerative and personalized medicine. However, their usefulness in studying epithelial transport and barrier function is limited by their convoluted 3D structure, which makes their lumen-facing apical side hard to access. Therefore, 3D organoids are often digested and seeded in 2D to form confluent organoid monolayers on permeable filter inserts called Transwells.

We previously established what we regard as the best-characterized human duodenum-derived organoid monolayer model to study epithelial barrier to date, where we analysed barrier properties by molecular, structural and advanced electrophysiological methods, in comparison to Caco 2 cells and human duodenum tissue [16]. Other studies using human intestinal organoid monolayers to study epithelial barrier and transport function relied on transepithelial resistance (TER) measurements, a simplistic assessment of barrier function and functional analysis of passive paracellular transport [17–27]. However, there is little to no data on active vectorial transport such as transcytosis, chloride secretion, and nutrient absorption, all essential physiological functions of the small intestinal mucosa. Furthermore, all prior studies were performed using Transwells coated with a gelatinous matrix such as BME for anchorage.

There are, however, several disadvantages of BME [28–30]. BME is extracted from purified Engelbreth-Holm-Swarm (EHS) mouse sarcoma tumours and therefore requires mass mice sacrifice. Furthermore, various formulations of BME are sold under several trade names such as Matrigel™, Cultrex™, Geltrex™ and others (collectively referred to here as BME); hence, manufacture-to-manufacturer as well as batch-to-batch variability of BME extracts limits reproducibility. Additionally, BME poorly recapitulates the basement membrane as the main component of the human intestinal basement membrane is collagen type IV [31], while laminin is the main component of BME [32]. Lastly, its mouse tumour extract origin is problematic as its composition is also not fully understood and thus, BME could have unknown mouse- and tumour-specific effects on human-derived organoids and perturbate their barrier and transport properties.

We hypothesized that the common practice of pre-coating Tranwells with BME to help cells adhere could hinder the diffusion of cytokines, ions, micro- and/or macromolecules along the apical and basolateral axis, and therefore optimized our previous protocol [16] to generate robust BME-free organoid monolayers that do not need a matrix for anchorage. Using monolayers derived from digested human duodenum-derived 3D organoids, we investigated whether these monolayers could polarize, form tight junctions and differentiate into major duodenum cell types. Furthermore, we functionally investigated their TER dynamics, cytokine responsiveness, transcytosis and paracellular barrier functions. We then validated their usefulness in studying transcellular transport function using conventional assays to measure chloride secretion, glucose and short-chain fatty acid (SCFA) uptake.

## Results

### TER growth dynamics of organoid monolayers

**Fig. 1:**
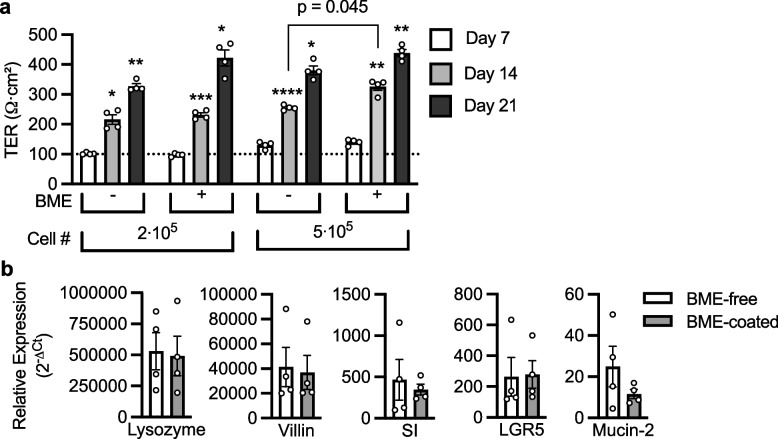
TER and cell markers of organoid monolayers. **a** 3D organoids of Patient 5C4 were split to single cell level and 2∙10^5^ or 5∙10^5^ cells were seeded on BME-free or BME-coated Transwells. TER was measured using chopstick electrodes. Duplicates from two independent experiments are shown. **b** Relative mRNA expression (normalized to GAPDH) of major duodenum cell markers is compared for BME-free or BME-coated organoid monolayers. SI: Sucrase-isomaltase, LGR5: Leucine-rich repeat-containing G-protein coupled receptor 5. *n* = 4 patient samples, in duplicates

We sought out to establish whether pre-coating Transwells with BME before seeding the single cells of our human duodenum-derived 3D organoids affected the growth dynamics of the monolayers. The presence or absence of BME pre-coating did not significantly affect the growth kinetics of the organoid monolayers except for monolayers seeded with 5∙10^5^ cells on day 14 where corrected TER values of BME-coated organoid monolayers were higher compared to BME-free (326.3 ± 12.5 Ω∙cm^2^ versus 255.3 ± 4.2 Ω∙cm^2^, respectively, *p* = 0.045, Fig. 1a). TER increased by at least 100 Ω∙cm^2^ weekly, irrespective of the cell number, presence or absence of BME pre-coating. Confluence was considered TER higher than 100 Ω∙cm^2^ (dotted line, Fig. 1a), a conservative estimate of the lowest TER value where apical medium volume consistently remained unchanged for at least 2 days, consistent with a previous report [23]. Confluence was reached by day 7 when 5∙10^5^ cells were seeded and day 14 if 2∙10^5^ cells were seeded. Similar growth dynamics were obtained for another patient sample; however, it is worth noting that this patient sample grew slower, with monolayers mostly reaching confluence by day 14 rather than day 7 (Additional File 1:Fig.S1). In an effort to increase the TER of the BME-free organoid monolayers, we seeded even higher cell densities of up to 4∙10^6^ cells and indeed, during the first 21 days, cell density was directly proportional to TER, after which the TER plateaued until the cultures were discontinued at day 49 (Additional File 1:Fig.S2).

### Phenotypic characterization of organoid monolayers

Next, we aimed to confirm the presence of the major duodenum cell types in our organoid monolayers. RT-qPCR data suggested an abundance of markers for Paneth cells (lysozyme) and enterocytes (villin and sucrase-isomaltase, SI) and modest expression of markers for stem cells (Leucine-rich repeat-containing G-protein coupled receptor 5, LGR5) and goblet cells (Mucin-2), with no significant difference in BME-coated and BME-free organoid monolayers due to large scatter (Fig. 1b). The presence of enteroendocrine cells was also investigated, although only two out of the four tested patient samples had detectable chromogranin A mRNA transcripts (Additional File 1:Fig. S3).

**Fig. 2:**
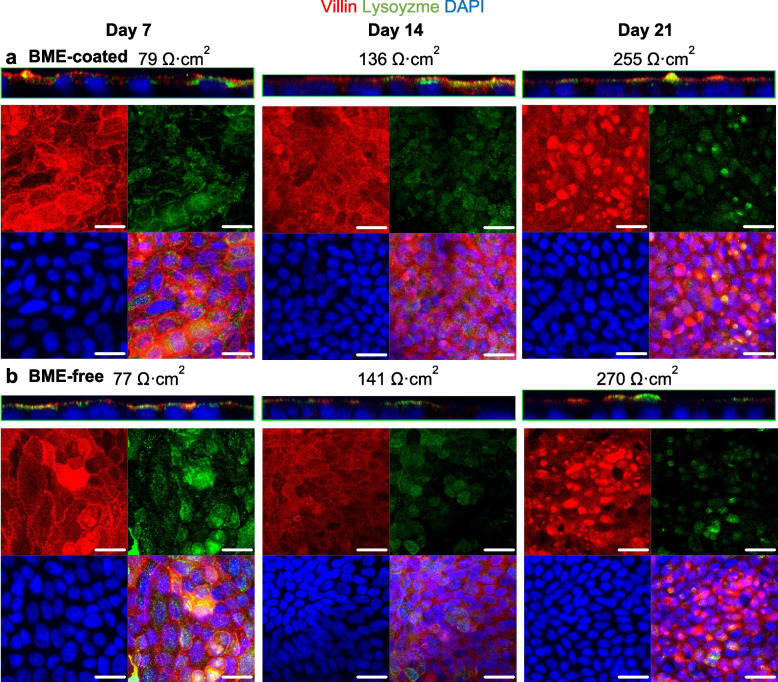
Villin and lysozyme staining of organoid monolayers over time. *Z*-axis and confocal projections of organoid monolayers seeded on **a** BME-coated or **b** BME-free Transwells, stained for differentiated cells (Villin) and Paneth cells (Lysozyme) on days 7, 14 and 21

**Fig. 3:**
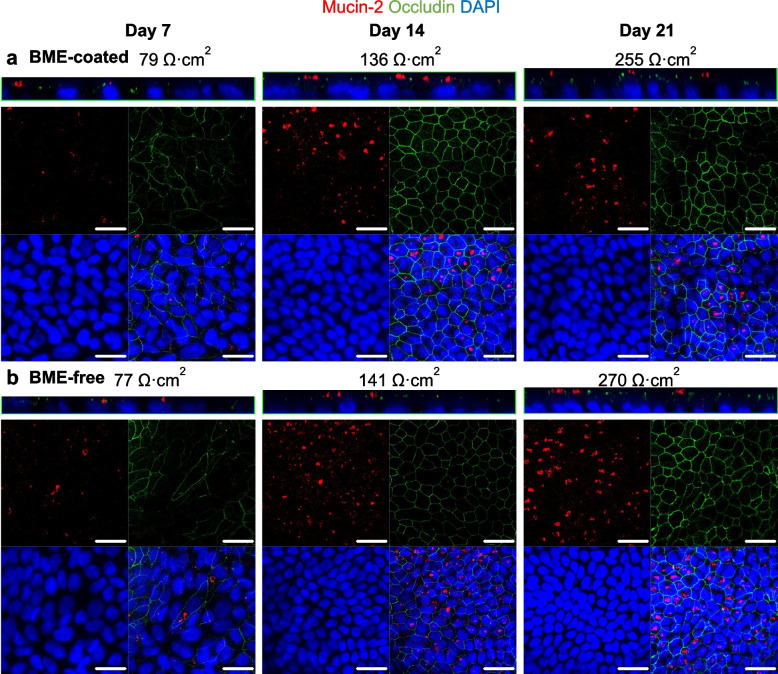
Mucin-2 and Occludin staining of organoid monolayers over time. *Z*-axis and confocal projections of organoid monolayers seeded on **a** BME-coated or **b** BME-free Transwells, stained for goblet cells (Mucin-2) and tight junction protein, Occludin on days 7, 14 and 21

The epithelial integrity, polarization and protein expression of major duodenum cell markers were investigated through immunofluorescence staining. The organoid monolayers consisted of villin-positive differentiated cells and lysozyme-positive Paneth cells from day 7 after cell seeding, with no obvious differences between BME-coated (Fig. 2a) and BME-free (Fig. 2b) monolayers. Villin staining localized to the brush borders of duodenum tissue (Additional File 1:Fig. S4) and organoid monolayers (Fig. 2a-b). Lysozyme-positive Paneth cells tended to group together, consistent with a previous report where organoid monolayers recapitulated the crypt-villus cell type segregations [33]. Mucin-2 staining was also detected within 7 days in BME-coated (Fig. 3a) and BME-free (Fig. 3b) monolayers, localized towards the apical side, recapitulating mucin secretion in duodenum tissue (Additional File 1:Fig. S4). Occludin staining became more robust from day 14 (Fig. 3a,b). The expression of other well-characterized tight junction proteins was further investigated in BME-free organoid monolayers, confirming the colocalization of Occludin with ZO-1 at the bicellular and Tricellulin at the tricellular junctions (Additional File 1:Fig. S5). With increasing TER, we could visually confirm a striking increase in cell number between day 7 and 14 as evidenced by the number of nuclei in each field of view (Figs. 2 and 3). Experiments were henceforth performed using monolayers at days 14–21, with 5∙10^5^ ± 1∙10^5^ cells initially seeded on each Transwell.

### Barrier and transport properties of organoid monolayers

**Fig. 4:**
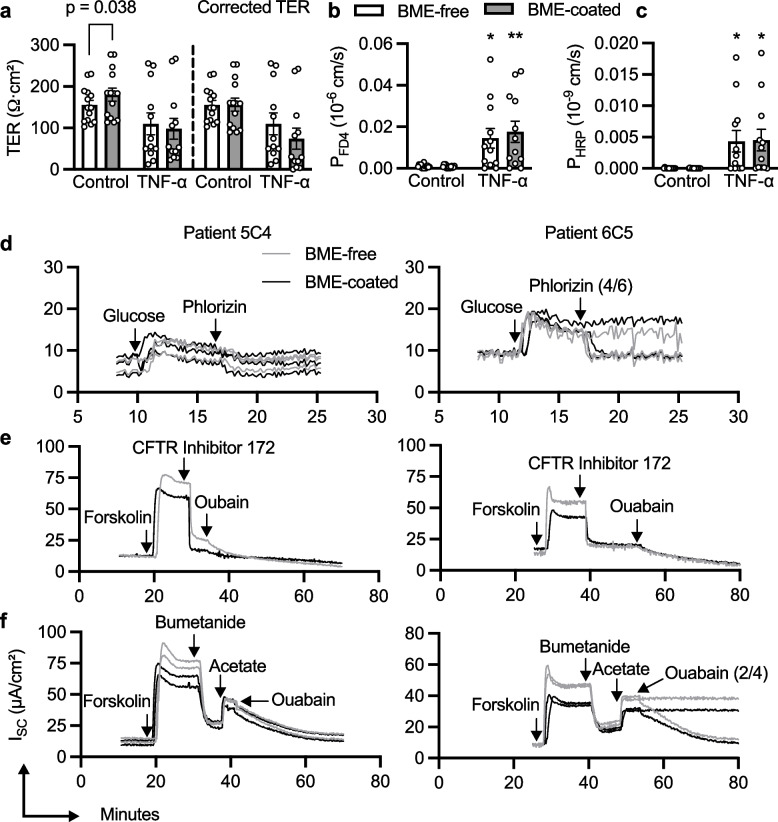
Functional analysis of organoid monolayers in Ussing chamber experiments. Effects of TNF-α on the **a** TER, **b** FD4 permeability and **c** HRP permeability, all monitored in parallel in Ussing chambers, of organoid monolayers seeded on BME-coated (grey) and BME-free (white) Transwells. *n* = 3 patient samples, each with 3–6 Transwells from 2–3 independent experiments. **d** Apical application of glucose (10 mM) increased short-circuit current (I_SC_), which was decreased by apical application of phlorizin (50 μM added to 4/6 chambers). **e** Apical application of forskolin (10 μM) increased I_SC_, which was decreased by the apical application of CFTR Inhibitor 172 (20 μM) and then further decreased by basolateral application of ouabain (500 μM). **f** Apical application of forskolin (10 μM) and sodium acetate (30 mM) increased I_SC_, which was decreased by the basolateral application of bumetanide (10 μM) and ouabain (500 μM added to 2/4 chambers), respectively. These experiments were all done on the same set of 3 BME-free (grey) and 3 BME-coated (black) Transwells, repeated for 2 patient samples

Next, we investigated whether BME-free organoid monolayers could model epithelial barrier dysfunction following TNF-α stimulation and whether the BME coat could hinder the movement of cytokines and macromolecules across the apical-basolateral axis. Unlike in Fig. 1a where *n* = 4 Transwells, after correcting for the contribution of the BME coat to the TER, there was no longer a significant difference between the TER of BME-coated and BME-free organoid monolayers (Fig. 4a, *n* = 14 Transwells). TNF-α tended to reduce the TER of BME-coated and BME-free organoid monolayers by 52.6 ± 16.1% and 29.3 ± 16.9%, respectively. Consequently, TNF-α made BME-coated and BME-free organoid monolayers comparably permeable to 4 kDa FD4 and 44 kDa HRP (Fig. 4b, c).

We also investigated whether transcellular transport functions of the duodenum were maintained by BME-free organoid monolayers and whether the movement of ions across the monolayers could be hindered by the BME coat. To this end, a range of pharmacologically active compounds with well-established biological responses were tested. Na^+^-glucose cotransporter 1 (SGLT1) is specifically expressed in the small intestine and it is activated by glucose and competitively inhibited by phlorizin [34, 35]. Forskolin increases short-circuit current (I_SC_) by stimulating cyclic adenosine monophosphate (cAMP)-dependent apical chloride secretion via the cystic fibrosis transmembrane regulator (CFTR) [36], a process that can be counteracted by several blockers including CFTR Inhibitor 172 [37], bumetanide—an inhibitor of basolateral Cl^−^ uptake through the Na^+^K^+^2Cl^−^ co-transporter (NKCC1) and ouabain, which blocks the basolateral Na^+^/K^+^-ATPase [38]. Ouabain also reduces I_SC_ elicited by HCO^−^_3_ secretion via apical SCFA/Na^+^-coupled monocarboxylate transporter-1 (SMCT1) [39].

Firstly, the effect of glucose and phlorizin on the I_SC_ was measured, revealing no obvious difference between BME-coated and BME-free organoid monolayers (Fig. 4d). I_SC_ stabilized to a baseline level of 6.7 ± 0.5 µA/cm^2^ for Patient 5C4 and 9.2 ± 0.4 µA/cm^2^ for Patient 6C5. Glucose increased the I_SC_ from baseline to an average maximum high of 12.3 ± 1.1 µA/cm^2^ for Patient 5C4 and 19.3 ± 0.4 µA/cm^2^ for Patient 6C5. As expected, phlorizin inhibited the glucose-induced I_SC_ increase and promptly returned the I_SC_ to baseline in all chambers, except for the two chambers of Patient 6C5 where phlorizin was not added to serve as controls. Next, the effect of forskolin and CFTR Inhibitor 172 on the I_SC_ was measured revealing subtle but consistent differences between BME-coated and BME-free organoid monolayers (Fig. 4e). I_SC_ stabilized to comparable baseline levels of 11.7 ± 0.8 µA/cm^2^ for all samples. As expected, forskolin increased the I_SC_ of Patient 5C4 by up to 70.4 ± 7.2 µA/cm^2^ for BME-free organoid monolayers compared to only 57.3 ± 4.9 µA/cm^2^ for BME-coated organoid monolayers and for Patient 6C5, 49.6 ± 6.6 µA/cm^2^ for BME-free organoid monolayers compared to only 29.7 ± 4.7 µA/cm^2^ for BME-coated organoid monolayers. Application of CFTR Inhibitor 172 and subsequent addition of ouabain was sufficient to return the I_SC_ to baseline for all samples. Bumetanide also decreased the forskolin-induced I_SC_, which stabilized at an average of 21.4 ± 2.4 µA/cm^2^ for all samples (Fig. 4f). Lastly, acetate, a SCFA, increased the I_SC_ of Patient 5C4 by up to 11.7 ± 1.2 µA/cm^2^ for BME-free organoid monolayers similar to 12.2 ± 4.4 µA/cm^2^ for BME-coated organoid monolayers and for Patient 6C5, 18.0 ± 1.1 µA/cm^2^ for BME-free organoid monolayers compared to only 13.8 ± 1.0 µA/cm^2^ for BME-coated organoid monolayers. During the entire course of the experiment, the TER of BME-coated organoid monolayers was higher than the TER of BME-free organoid monolayers (Additional File 1:Fig. S6), mirroring the modest but consistent I_SC_ differences between BME-coated and BME-free organoid monolayers.

## Discussion

The intestinal barrier has been primarily studied using transformed human colon-derived cell lines such as Caco2, T84 and HT-29 [9–11] or BME-coated organoid monolayers [16–27], despite their disadvantages discussed earlier. Small intestinal cell lines are scarce with HuTu-80 [40, 41] being the only human duodenum-derived cell line and pig jejunum-derived IPEC-J2 [42] thus also being used. Unlike cell lines, organoid monolayers recapitulated duodenal cellular composition and three independent transcellular transport pathways: chloride secretion, SCFA uptake and glucose absorption, the latter absent in HuTu-80 [40], IPEC-J2 [42] and HT-29 cells [43]. Forskolin-induced I_SC_ of BME-free organoid monolayers was 8 times higher than of Caco-2 and HT-29-MTX cells [43], and modestly but consistently higher than of BME-coated organoid monolayers. Our previous work showed that organoid monolayers expressed bicellular (claudins 1–5 and 7) and tricellular tight-junction proteins (tricellulin, angulin-1 variant α and variant β) to maintain the epithelial barrier [16]; we show here that TNF-α perturbed this barrier, rendering organoid monolayers permeable to 4 kDa FD4 and 44 kDa HRP, well-characterized markers of paracellular transport [44] and transcytosis [45], respectively. BME-free organoid monolayers are thus an improved model for studying transcytotic, paracellular and especially transcellular transport.

Despite not having a BME coat for anchorage, BME-free organoid monolayers had stable TER for weeks, making them suitable for studying the epithelial barrier over long periods. TER growth dynamics varied between patient samples suggesting that human-to-human heterogeneity is sustained in our organoid model. Organoid monolayers contained 4–5 major duodenum cell markers with large scatter in mRNA expression further highlighting conserved patient sample heterogeneity. Glucose absorption is a small intestine-specific function, thus showing that duodenal transport function is also maintained in our model. The presence of BME coating did not affect macromolecular permeability but modestly hindered the diffusion of small molecules like chloride ions, when inducing high transport rates. Using chopstick electrodes, we measured the contribution of the BME coat to the TER to be 24 Ω⋅cm^2^. Even after correcting for this, BME-coated organoid monolayers at times had higher TER than BME-free, but significance waned as sample size increased, and this could be counteracted by seeding higher cell numbers. Using much more advanced impedance spectroscopy, we previously showed that 2% (v/v) BME coat increases subepithelial resistance of organoid monolayers by approximately 36 Ω⋅cm^2^ [16], where again, only modest chloride secretion was seen in BME-coated organoid monolayers. Since it is known that increasing series resistances leads to underestimation of active transport by the measured I_SC_ [46], the extra resistance conferred by the BME layer may explain the more robust chloride secretion in BME-free organoid monolayers.

Pre-coating Transwells with gelatinous matrices such as gelatine, collagen or BME has been an indispensable step in previous protocols [16–27, 47, 48]. Initially, 3D organoids used to be partially digested into fragments or cell clumps using Trypsin [17–19, 47]. TrypLE™ Express, a less toxic Trypsin alternative [49], became more popular as it more reliably digests 3D organoids into single cells [20–22, 26, 48, 50, 51]. However, cells undergo anoikis or apoptosis when they lose their cell–matrix and/or cell–cell interactions [52, 53], which is mitigated by the use of gelatinous matrix pre-coating agents and/or seeding partially digested organoids, respectively. Efforts to mitigate anoikis in our protocol included seeding high cell numbers of at least 5∙10^5^ cells per Transwell (8∙10^5^ cells per cm^2^) to increase cell-to-cell interactions and using a Rho kinase inhibitor during the first 2 days after seeding single cells to block caspase-dependent apoptotic pathways [54, 55]. While others have used up to 2–5% (v/v) BME to pre-coat their Transwells before seeding their intestinal organoid-derived single cells [20–22, 24], van der Hee et al. achieved confluent monolayers after pre-coating Transwells with almost negligible 0.5% (v/v) BME [48]. We speculate that, as was shown by Kauffman et al. who generated the first organoid monolayers in 2013 [17], partially digested organoid fragments and cell clumps need a gelatinous matrix to adhere to, flatten out and homogenize into a monolayer, but BME pre-coating is no longer necessary when seeding single cells if other efforts to mitigate anoikis are taken.

While our protocol negates the need for BME in 2D organoid culture, BME was used in our 3D organoid culture due to its ease of use and commercial accessibility. However, given the disadvantages of BME discussed earlier, decellularized extracellular matrix derived from human donors, customizable and well-defined synthetic hydrogels, or natural hydrogels approved for clinical use such as collagen could have been used as alternatives [28–30]. Previously, organoid monolayers could be passaged on collagen, but not BME-coated surfaces, rendering tedious 3D organoid culture dispensable [50, 56]. Our BME-free organoid monolayers reached confluence but could not be serially passaged in cell culture flasks (unpublished data). Those monolayers contained mostly undifferentiated cells [50, 56] while ours contained mostly differentiated cells as the stem cell signalling growth factor, Wnt, was absent in our 2D medium. This could also explain why our monolayers could not be passaged, as stem cells have a higher self-renewal capacity than differentiated cells.

## Conclusions

We are the first to characterize BME-free organoid monolayers in detail, showing that, unlike transformed cell lines, BME-free organoid monolayers recapitulated duodenal cellular composition and multiple independent important transcellular transport pathways. Furthermore, we show that pre-coating Transwells with BME is an archaic practice that introduces an unnecessary diffusion barrier. Therefore, compared to existing organoid models, BME-free organoid monolayers represent an improved model for studying transcytotic, paracellular but especially transcellular transport. As BME is extracted from mice, our protocol brings us one step closer to making organoid culture more animal-free, in line the 3Rs principle.

## Methods

### Samples

This study was performed in compliance with the Ethics Committee of Charité-Universitätsmedizin Berlin (EA4/015/13). Four individuals, two males and two females aged 30 to 37 years, were included in this study (Patients 1C1, 3C2, 5C4, and 6C5). All were scheduled to undergo routine endoscopy and gave written informed consent to be included in this study. Up to five duodenal biopsy specimens were obtained with 3.4-mm biopsy forceps from each patient and organoids were generated immediately. Experiments were performed using at least two of the fastest-growing patient samples.

### 3D organoid culture

Organoids were generated and propagated using the protocol from Bartfeld et al. with minor modifications [57]. Briefly, biopsies were cut into small pieces with scissors, washed with ice-cold phosphate-buffered saline (PBS, Gibco, Thermo Fisher, Waltham, USA) five times, and then dissociated with 2.5 mM ethylenediaminetetraacetic acid solution (EDTA, Merck, Darmstadt, Germany) in PBS for up to 10 min at room temperature. The crypts were liberated in a Petri dish by pressing a microscope slide down above the pieces using enough force until the liquid surrounding the biopsies turned cloudy, after which the liberated crypts were confirmed by observation under a brightfield light microscope. The supernatant containing the crypts was then pelleted at 500 × *g* for 5 min at 4 °C. The crypts were cultured in pre-warmed 24-well plates (TPP/Merck, Darmstadt, Germany), suspended in 50 μL Cultrex™ BME (R&D Systems, Minneapolis, USA). The cultures were then fed 3D medium containing 10 μM Rho kinase inhibitor (Y-27632, Tocris, Bristol, UK) for 2 days, with the medium being replenished every 2 to 3 days. The 3D medium consisted of Advanced Dulbecco’s modified Eagle’s medium (DMEM)/F12 (Gibco) conditioned medium that was produced from stable transfected HEK293 cells (ATTC, Virginia, USA) with 50% (v/v) L-WRN [58], 20% (v/v) Rspo1 [59], 10% (v/v) Noggin [60] supplemented with 50 ng/ml human recombinant epithelial growth factor (EGF, Peprotech, Hamburg, Germany), 1 mM N-acetylcysteine (NAC, Sigma-Aldrich, Steinheim, Germany), 10 mM nicotinamide (NIC, Sigma-Aldrich), 1 μM p38 inhibitor (SB-202190, Stemcell, Vancouver, Canada), 500 nM TGF-β inhibitor (A83-01, Stemgent, Cambridge, USA), 1 × B27 (Gibco), and 1 × N2 (Gibco). Organoids were passaged an average of 1:4 wells weekly. Briefly, after collecting the BME containing the organoids in 15-mL falcon tubes, the BME was washed off using ice-cold Advanced DMEM/F12 containing 10 mmol/L HEPES (Gibco), 1 × Glutamax (Gibco), and 1% 100 U/ml penicillin and 100 mg/ml streptomycin (Penicillin–Streptomycin Solution, Corning, New York, USA), henceforth referred to as AD + + + . The organoid pellet was mechanically dissociated by re-suspension using a 22-gauge needle and 1-mL syringe in 2 mL ice-cold AD + + + . The organoid pieces were washed in AD + + + and re-cultured in BME as described above.

### 2D organoid culture

**Fig. 5:**
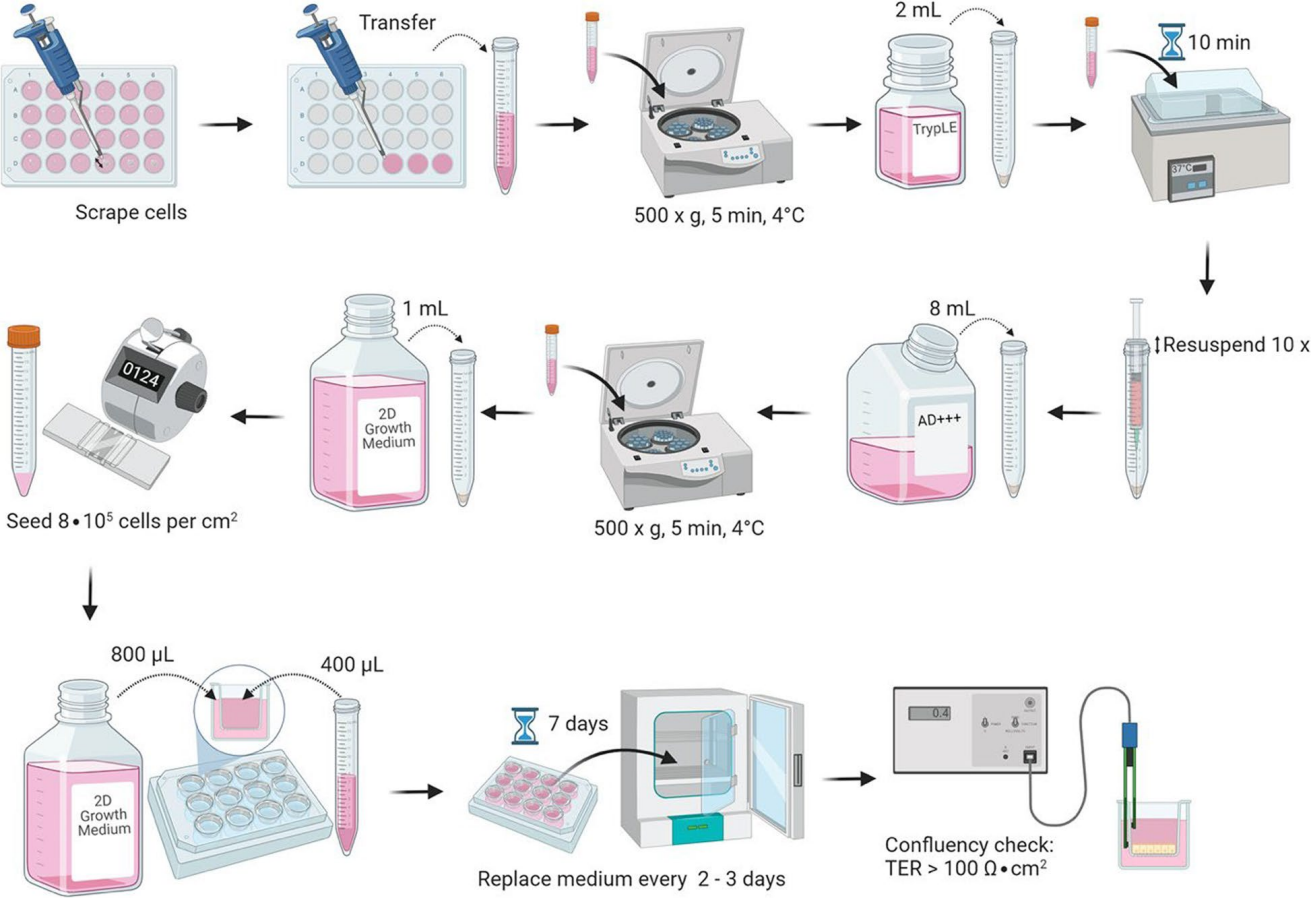
Generating organoid monolayers. 3D organoids in BME are collected and washed before being enzymatically and mechanically digested to single cells. The single cells are then washed and counted before being seeded on Transwells to form monolayers

3D organoids were digested to single cells to generate 2D organoids or organoid monolayers as illustrated (Fig. 5). Organoids were collected and washed as described above, and the organoid pellet was enzymatically digested in 2 mL TrypLE™ Express for 10 min in a 37 °C water bath. Organoids were further mechanically dissociated to single cell level by re-suspending 10 times using the sheer force of a 22-gauge needle and 1-mL syringe. During this step, the needle tip was placed as close as possible to the side of the falcon tube and tilted so that the opening of the needle faced the tube. This was done to create a whirlpool liquid flow to maximize agitation while preventing excessive air bubble formation. Cells were washed with ice-cold AD + + + , and potential cell clumps dissociated with a needle and syringe once again before cell counting. For coating, 100 μl of 2% (v/v) BME in ice-cold AD + + + was added to each Transwell permeable filter insert (PIHP01250, Millipore, Schwalbach, Germany). Transwells were incubated for at least 1 h at 37 °C and unsolidified AD + + + was removed before cell seeding. Optimally, 5∙10^5^ cells were seeded on each Transwell and topped up to 400 μL apically and 800 μL basolaterally with 2D medium containing 10 μM Rho kinase inhibitor for 2 days and then, every 2 to 3 days without the Rho kinase inhibitor. 2D medium was identical to the 3D medium in composition and concentrations except it was without L-WRN, SB-202190 and A83-01. TER was monitored using a chopstick electrode (STX2, World Precision Instruments, Sarasota, USA) and corrected by subtracting the resistance of cell-free transwells with (170 Ω) or without (130 Ω) BME coating, multiplied by the effective area of the Transwells (0.6 cm^2^).

### Quantitative real-time PCR (RT-qPCR)

Total RNA was extracted with NucleoSpin™ RNA/Protein Purification Mini Kit (Macherey–Nagel, Düren, Germany) according to the manufacturer’s protocol. A Nanodrop 1000 (Thermo Fisher) was used to quantify RNA and 1.5 µg RNA was reverse transcribed with the High-Capacity RNA-to-cDNA Kit (Applied Biosystems/Thermo Fisher). RT-qPCR reactions were performed in a thermocycler (QuantStudio 3, Applied Biosystems) using 3 μg of cDNA template, 1 μL of the desired probe, 10 μL of RT-qPCR Master Mix (Applied Biosystems) and nuclease-free water to a final volume of 20 μl. The following human probes used were all from Thermo Fisher: Leucine-rich repeat-containing G-protein coupled receptor 5 (LGR5, Hs00969422_m1), Mucin-2 (MUC2, Hs00894025_m1), Lysozyme (LYZ, Hs00426232_m1), Villin (VIL1, Hs01031739_m1), Sucrase isomaltase (SI, Hs00356112_m1) and Chromogranin A (CHGA, Hs00900370_m1). Data were analysed according to the 2^ΔCT^ method [61], normalized to GAPDH (Applied Biosystems).

### Immunofluorescence staining

To identify the cell markers at protein level, organoid monolayers and duodenum tissue sections were stained with antibodies according to a previous publication [33]. Briefly, monolayers were fixed with 4% paraformaldehyde (Electron Microscopy Sciences, Hatfield, USA) at room temperature for 15 min. Tissue sections were sliced, deparaffinized and heated in sodium citrate buffer (pH 6.0) for epitope retrieval. Tissue and monolayers were permeabilized with 1% Triton X-100 (Serva, Heidelberg, Germany) in PBS at 4 °C for 15 min, blocked with 3% bovine serum albumin (BSA, Sigma-Aldrich, Steinheim, Germany) and 0.1% Triton X-100 in PBS, and incubated at 4 °C overnight with primary antibodies. The primary antibodies used were goat anti-Villin (1:100, SC-7672, Santa Cruz), rabbit anti-lysozyme (1:200, F0372, Dako), rabbit anti-Mucin-2 (1:300, SC-15334, Santa Cruz), rabbit anti-Occludin (1:100, 71–1500, Invitrogen), mouse anti-Occludin (1:100, 33–1500, Invitrogen), rabbit anti-Tricellulin (1:100, 700,191, Invitrogen) and mouse anti-Zonula Occludens-1 (ZO-1, 1:100, 610,966, BD Biosciences). After rigorous washing, secondary antibodies were applied for 1 h at room temperature. These were goat anti-rabbit Alexa Fluor 488 (1:1 000, A11034, Invitrogen) and goat anti-mouse Alexa Fluor 594 (1:1 000, A11032, Invitrogen), as well as nuclei stain 4’, 6-diamidino-2-phenylindole (DAPI, 1:1 000 of a stock solution of 1 mg/ml in methanol, 10 236 276 001, Roche). Images were obtained using a confocal laser scanning microscope (LSM 780, Carl Zeiss AG, Jena, Germany).

### Ussing chamber experiments

Confluent organoid monolayers were mounted in Ussing chambers for electrophysiological analysis. The bathing solution (pH 7.4) contained 140 mM Na^+^, 123.8 mM Cl^−^, 5.4 mM K^+^, 1.2 mM Ca^2+^, 1.2 mM Mg^2+^, 2.4 mM HPO_4_^2−^, 0.6 mM H_2_PO_4_^−^, 21 mM HCO_3_^−^ and 10 mM D-(+)-glucose. The same bathing solution was applied on the apical and basolateral side to eliminate chemical gradients. The solution was kept in circulation by 95% O_2_/5% CO_2_ gas movement and the temperature was kept constant at 37 °C using temperature-controlled water reservoirs. Short-circuit current (I_SC_ in µA/cm^2^) of two patient samples seeded on BME-coated and BME-free (three of each, respectively) was continuously measured. Three separate experiments were conducted on the same sets of Transwells. In the first experiment which occurred within the first 25 min, the effect of 10 mM glucose (Carl Roth, Karlsruhe, Germany) and 50 μM phlorizin (Sigma) on the I_SC_ was measured. Next, the effect on I_SC_ following application of 10 μM forskolin (Cayman Chemical Company, Ann Arbor, USA), 20 μM cystic fibrosis transmembrane conductance regulator Inhibitor 172 (CFTR Inhibitor 172, Sigma) or 10 μM bumetanide (Sigma), and 500 μM ouabain (Merck) was also measured. And finally, the effect of 30 mM sodium acetate (Fluka, Buchs, Switzerland) on I_SC_ was measured. All substances were applied apically, except for bumetanide and ouabain, which were applied basolaterally.

### Permeability measurements

Confluent monolayers were pre-stimulated with 1 ng/mL human interferon-gamma (IFN-γ, Peprotech) for 3 days to upregulate tumour necrosis factor-alpha (TNF-α) receptors [62, 63], and then stimulated with 5 ng/mL human TNF-α (TNF-α, Peprotech) for up to 5 h until the TER reduced by up to 50% to around 130–200 Ω∙cm^2^. Permeability measurements were also performed in Ussing chambers under the conditions described above. The apical bathing solution contained 0.4 mM dialyzed FITC-labeled 4-kDa dextran (FD4, TdBConsultancy, Sweden) and 25 nM 44-kDa Horse Radish Peroxidase (HRP, Sigma). Permeability to FD4 and HRP was measured by collecting samples from the basolateral compartment at 0, 30, 60 and 90 min. HRP was quantified using a fluorogenic peroxidase substrate kit (Quanta Blu™, Thermo Fisher) and, along with FD4, detected fluorometrically using a Tecan Infinite M200 reader (Tecan, Switzerland). Permeabilities were calculated as the ratio of flux J (mol∙h^−1^∙cm^−2^) over concentration gradient Δc (mol/L).

### Statistics

Microsoft Excel version 16 (Microsoft) and/or Prism version 10 software (GraphPad) were used for graphing and statistical analysis. Results are given as mean ± SEM. Univariate analysis was performed using the 2-tailed Student’s *t*-test, while multivariate analysis was performed using the one-way or two-way ANOVA. Bonferroni–Holm adjustment was used for post hoc analysis in multiple testing. *P*-values < 0.05 were considered significant.

## Supplementary Information

Additional file 1: Figures S1-S6. Fig. S1 3D organoids of Patient 6C5 were digested to single cell level and 2∙10^5^ or 5∙10^5^ cells were seeded on BME-free or BME-coated Transwells. TER was measured using chopstick electrodes. Duplicates from two independent experiments are shown. Fig. S2 3D organoids were digested to single cell level and 1∙10^6^—4∙10^6^ cells were seeded on BME-free Transwells. Duplicates from one patient sample are shown. Fig. S3 Cycle threshold (CT) values of organoid monolayers following RT-qPCR or major duodenum cell markers. *n* = 4 patient samples. Fig. S4 Confocal snapshot of paraffin-embedded duodenum tissue section showing the location of major duodenum cell markers. Scale bars, 20 μm. Fig. S5 Confocal snapshots of tight junction protein expression of BME-free organoid monolayers. Scale bars, 20 μm. Fig. S6 Changes in TER following apical application of forskolin (10 μM), apical application of CFTR Inhibitor 172 (20 μM) or basolateral application of bumetanide (10 μM), apical application of sodium acetate (30 mM) and basolateral application of ouabain (500 μM).

## Data Availability

The datasets used and/or analysed during the current study are available from the corresponding author on reasonable request.

## Abbreviations

BME: Basement membrane extract
BSA: Bovine serum albumin
cAMP: Cyclic adenosine monophosphate
CFTR: Cystic fibrosis transmembrane regulator
DAPI: 6-Diamidino-2-phenylindole
DMEM: Dulbecco’s modified Eagle’s medium
EDTA: Ethylenediaminetetraacetic acid solution
EGF: Epithelial growth factor
EHS: Engelbreth-Holm-Swarm
FD4: Fluorescein Isothiocyanate-labeled 4 kDa dextran
HIV: Human immunodeficiency virus
HRP: Horse radish peroxidase
IBD: Inflammatory bowel disease
IFN-γ: Interferon-gamma
I_SC_: Short-circuit current
LGR5: Leucine-rich repeat-containing G-protein coupled receptor 5
NAC: N-acetylcysteine
NKCC1: Na^+^K^+^2Cl^−^ co-transporter
PBS: Phosphate-buffered saline
SCFA: Short-chain fatty acid
SGLT1: Sodium-glucose cotransporter 1
SI: Sucrase-isomaltase
SMCT1: Na^+^-coupled monocarboxylate transporter-1
TER: Transepithelial resistance
TNF-α: Tumour necrosis factor-alpha
