# Supplementary material for: Matrix-free human 2D organoids recapitulate duodenal barrier and transport properties
Source: BMC Biol. 2025 Jan 5;23:2. doi: 10.1186/s12915-024-02105-7 (PMC11702212; doi:10.1186/s12915-024-02105-7)
Supplement: Supplementary file 1 — Additional file 1: Figures S1-S6. Fig. S1 3D organoids of Patient 6C5 were digested to single cell level and 2∙105 or 5∙105 cells were seeded on BME-free or BME-coated Transwells. TER was measured using chopstick electrodes. Duplicates from two independent experiments are shown. Fig. S2 3D organoids were digested to single cell level and 1∙106—4∙106 cells were seeded on BME-free Transwells. Duplicates from one patient sample are shown. Fig. S3 Cycle threshold (CT) values of organoid monolayers following RT-qPCR or major duodenum cell markers. n = 4 patient samples. Fig. S4 Confocal snapshot of paraffin-embedded duodenum tissue section showing the location of major duodenum cell markers. Scale bars, 20 μm. Fig. S5 Confocal snapshots of tight junction protein expression of BME-free organoid monolayers. Scale bars, 20 μm. Fig. S6 Changes in TER following apical application of forskolin (10 μM), apical application of CFTR Inhibitor 172 (20 μM) or basolateral application of bumetanide (10 μM), apical application of sodium acetate (30 mM) and basolateral application of ouabain (500 μM). [file 12915_2024_2105_MOESM1_ESM.docx]

**Additional File 1**

**Fig. S1** 3D organoids of Patient 6C5 were digested to single cell level and 2∙10^5^ or 5∙10^5^ cells were seeded on BME-free or BME-coated Transwells. TER was measured using chopstick electrodes. Duplicates from two independent experiments are shown.

**Fig. S2** 3D organoids were digested to single cell level and 1∙10^6^ - 4∙10^6^ cells were seeded on BME-free Transwells. Duplicates from one patient sample are shown.

**Fig. S3** Cycle threshold (CT) values of organoid monolayers following RT-qPCR or major duodenum cell markers. n = 4 patient samples.

**Fig. S4** Confocal snapshot of paraffin-embedded duodenum tissue section showing the location of major duodenum cell markers. Scale bars, 20 μm


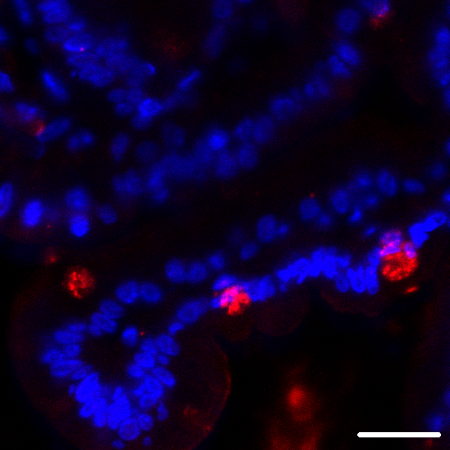


Villin Lysozyme DAPI

Mucin-2 DAPI


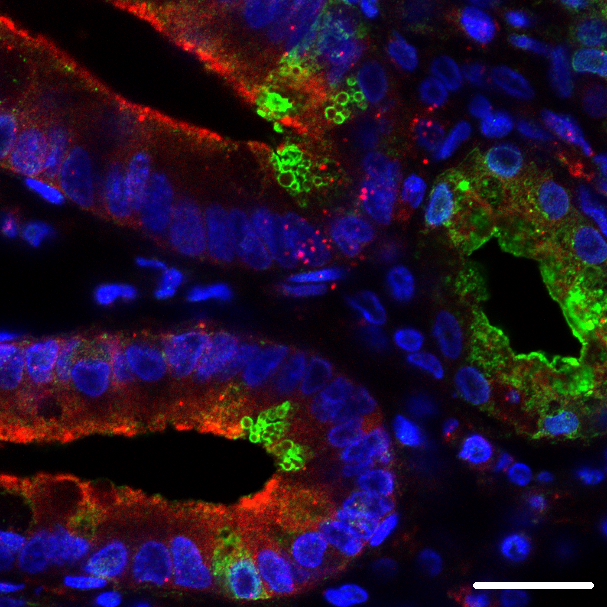


**Fig. S5** Confocal snapshots of tight junction protein expression of BME-free organoid monolayers. Scale bars, 20 μm

ZO-1 Occludin DAPI

Occludin Tricellulin DAPI


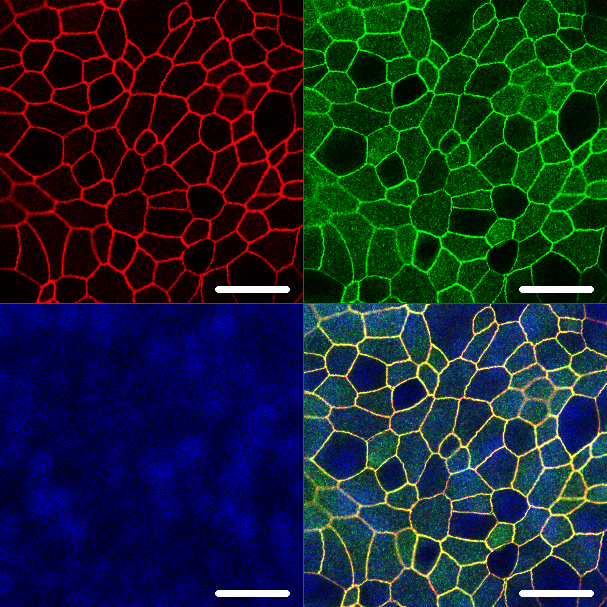

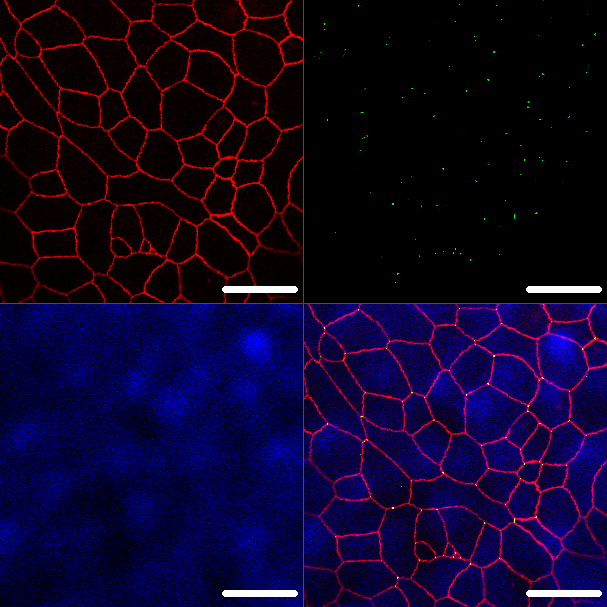


**Fig. S6** Changes in TER following apical application of forskolin (10 μM), apical application of CFTR Inhibitor 172 (20 μM) or basolateral application of bumetanide (10 μM), apical application of sodium acetate (30 mM) and basolateral application of ouabain (500 μM).
